# Plant-to-Plant Variability in Root Metabolite Profiles of 19 *Arabidopsis thaliana* Accessions Is Substance-Class-Dependent

**DOI:** 10.3390/ijms17091565

**Published:** 2016-09-16

**Authors:** Susann Mönchgesang, Nadine Strehmel, Diana Trutschel, Lore Westphal, Steffen Neumann, Dierk Scheel

**Affiliations:** 1 Leibniz Institute of Plant Biochemistry, Stress and Developmental Biology, Weinberg 3, 06120 Halle (Saale), Germany; nadine.strehmel@ipb-halle.de (N.S.); diana.trutschel@ipb-halle.de (D.T.); lore.westphal@ipb-halle.de (L.W.); steffen.neumann@ipb-halle.de (S.N.); 2 Institute of Computer Science, Martin-Luther-University Halle-Wittenberg, Von-Seckendorff-Platz 1, 06120 Halle (Saale), Germany; 3 German Center for Neurodegenerative Disesaes, Stockumer Straße 12, 58453 Witten, Germany

**Keywords:** LC/MS, GC/MS, *Arabidopsis*, secondary metabolism, natural variation, individual variability, metabolite profiling

## Abstract

Natural variation of secondary metabolism between different accessions of *Arabidopsis thaliana* (*A. thaliana*) has been studied extensively. In this study, we extended the natural variation approach by including biological variability (plant-to-plant variability) and analysed root metabolic patterns as well as their variability between plants and naturally occurring accessions. To screen 19 accessions of *A. thaliana*, comprehensive non-targeted metabolite profiling of single plant root extracts was performed using ultra performance liquid chromatography/electrospray ionization quadrupole time-of-flight mass spectrometry (UPLC/ESI-QTOF-MS) and gas chromatography/electron ionization quadrupole mass spectrometry (GC/EI-QMS). Linear mixed models were applied to dissect the total observed variance. All metabolic profiles pointed towards a larger plant-to-plant variability than natural variation between accessions and variance of experimental batches. Ratios of plant-to-plant to total variability were high and distinct for certain secondary metabolites. None of the investigated accessions displayed a specifically high or low biological variability for these substance classes. This study provides recommendations for future natural variation analyses of glucosinolates, flavonoids, and phenylpropanoids and also reference data for additional substance classes.

## 1. Introduction

Metabolomics is one of the “-omics” disciplines in plant science. With the help of hyphenated techniques such as gas chromatography coupled to mass spectrometry (GC/MS) or liquid chromatography-coupled mass spectrometry (LC/MS), a large spectrum of small molecules within a plant can be analysed. *Arabidopsis thaliana* (*A. thaliana*) is a model species to investigate secondary metabolic pathways. Naturally occurring accessions and their distinct phenotypes have evolved in different habitats and full genome sequencing revealed a substantial number of single nucleotide polymorphisms [1]. Compared to seeds and shoots, root metabolism is not as well investigated, but in plants it is crucial in order to provide the molecular building blocks for physical anchorage in the ground and to regulate all belowground processes. By root exudation, plants also communicate with their surrounding rhizosphere and soil microorganisms. In general, due to the relatively low biomass of *Arabidopsis*, especially in roots, material of several plants is pooled before sample preparation. With increasing sensitivity and decreasing costs of analytical techniques, pooling does not seem to be technically necessary anymore. Indeed, in some cases it is interesting to focus on individual variability to investigate which mechanisms determine plant metabolism without stress exposure. Once the plant material is pooled, the information on individual plants is irreversibly lost. Vice versa, smart experimental design allows for both—investigating variances on different levels (replicates) and detecting differences between accessions.

Several metabolomics studies examined the contribution of different variance sources to the total observed variance [2,3]. For nuclear magnetic resonance (NMR) metabolomics, Lewisetal et al. [2] found that extraction and instrumental deviations accounted for less than 10% and 1%, respectively, of the total variance in leaves of the accession L*er*-0. The substantial plant-to-plant variability of 52% in L*er*-0 could be reduced by pooling several plants to facilitate the separation of L*er*-0 from Col-0 samples. Reducing biological variability by pooling might allow for the fast detection of the effect of interest but nevertheless, it might miss subtle between-plant effects. Similar trends for extraction and instrumental variance were observed in comprehensive LC/MS-based metabolomics studies of Col-0 shoots [3]. Trutschel et al. [3] also provide a solution for how to incorporate different kinds of replicates into a powerful experimental design without the need for sample pooling.

Previous studies have investigated plant-to-plant variability during leaf development. The area of leaf six varied substantially between plants of the isogenic accession Col-0 at the same developmental stage, and this variability seems to converge in mature leaves [4]. Li et al. [5] determined there was 33%–40% plant-to-plant variability between the oil content of Col-0 seeds, and pointed out that this fact needs to be considered to draw statistically valid conclusions.

Plant-to-plant variability has neither been investigated in root metabolism nor have previous studies incorporated more than two *A. thaliana* accessions into a comprehensive root metabolic profiling analysis. Here, we analysed root metabolic profiles of 19 accessions, which were the founders of the multiparent advanced generation inter-cross (MAGIC) collection of *A. thaliana* [1,6], using a single-plant setup in a hydroponic system.

The aim of this study was to decompose the total variance of root metabolite profiles observed in untreated plants into the components attributable to (1) natural variation between accessions; (2) experimental batch; and (3) individual variability between plants. Furthermore, we investigated the relative biological variability of three important substance classes: glucosinolates (GSLs), flavonoids, and phenylpropanoids including oligolignols which seem to play a vital role in root (but not shoot) metabolism. Following the analysis of 19 accessions in their entirety, the variability of each accession was analysed to identify any particular highly or lowly variable accessions.

## 2. Results

### 2.1. Variability between Plants Is a Greater Source of Variance than Natural Variation between Accessions

Many studies on natural variation are primarily interested in differences between the accessions, and reduce plant-to-plant variability by pooling material to obtain fast results. However, to obtain a comprehensive picture of variability, the variance at each level of the experimental design should be incorporated.

**Figure 1:**
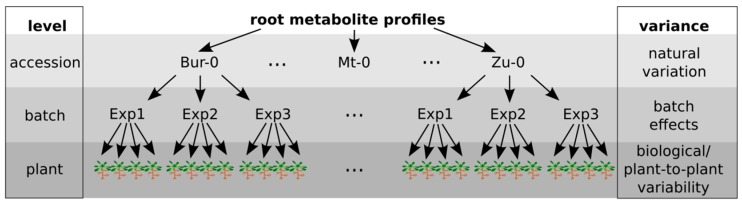
Nested experimental design with three levels. Each variance level had multiple replicates—to assess natural variation, 19 accessions of *Arabidopsis thaliana* (*A. thaliana*) were grown. Three independent biological experiments were performed to estimate non-biological variance derived from the experimental batch. To assess individual variability, four plants were harvested in each biological experiment for each accession. Single-plant root extracts were subjected to liquid chromatography-coupled mass spectrometry (LC/MS) and gas chromatography-coupled mass spectrometry (GC/MS) analysis.

The experimental setup of our study, shown in Figure 1, resulted in 222 single-plant LC/MS measurements in each electrospray ionization (ESI) mode. The alignment of chromatograms and spectra over 222 samples was performed, deviations in retention time (RT) and mass-to-charge ratio (*m*/*z*) were small across all samples (Figure S1) reflecting a sufficient quality of the measurements to analyse the effects of accession, experimental batch, and individual plant. Linear mixed models with all experimental levels as random effects were applied to decompose the total metabolic variance.

**Figure 2:**
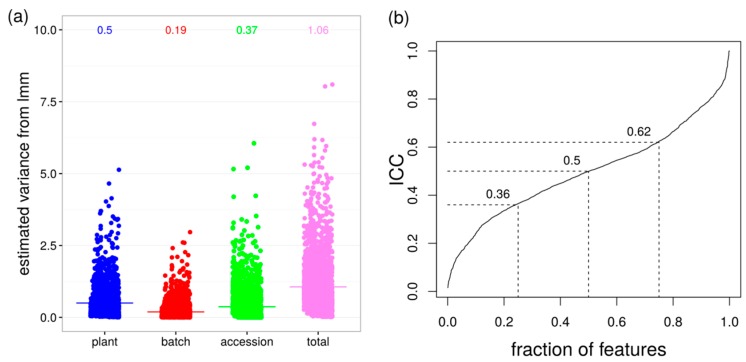
Variance decomposition of LC/electrospray ionization (ESI)(−) MS data set. (**a**) Variances for plant, batch and accession were estimated with a linear mixed model (lmm), dot—variance of one feature, bar and number—mean variance over 2730 features; (**b**) cumulative intraclass correlation (ICC) distribution for all features (σ^2^_plant_/σ^2^_total_), dotted lines indicate 25%, 50% and 75% quantiles.

The non-targeted metabolic profiles of the 19 accessions indicated that the between-accession variance is smaller than the plant-to-plant-variability over all features. The results for ESI(−) are shown in Figure 2a and for ESI(+) in Supplementary Figure S2.

The mean between-plant variance σ^2^_plant_ = 0.50 is 20% larger than the between-accession variance σ^2^_accession_ = 0.37. The estimated mean between-experiment variation σ^2^_batch_ = 0.19 is less than 40% of σ^2^_plant_. On average, plant-to-plant variability contributes to approximately half of the total variance (σ^2^_plant_/σ^2^_total_ = 0.47). However, this biological variance has to be interpreted in the context of the total variance for comparisons across features and platforms, i.e., knowing whether the feature with the highest σ^2^_plant_ also exhibits large σ^2^_total_. It may also occur that a feature with high σ^2^_plant_ has low σ^2^_total_, which determines the experimental design to include more replicates on the plant level in a potential validation study.

The intraclass correlation (ICC) according to Sampson et al. [7], here σ^2^_plant_/σ^2^_total_, reflects which fraction of total variance is attributable to the single plant and thus, a relative biological variability. The mean ICC ≈ 0.5 of a data set could either be representative for the majority of features (narrow interquartile range) or only for a few features if the interquartile range is broad. Figure 2b shows the cumulative ICC distribution over all features, with the fraction of features (*x*-axis) in increasing ICC (*y*-axis) order. The distribution revealed that 25%, 50%, and 75% of all these features had an ICC up to 0.36, 0.50, and 0.62. This implies that for half of the features, the plant-to-plant variability contributes to less than 50% to the total variance, and for the other half this variance level explains more than 50% of the total variance. In summary, in our non-targeted analysis of root metabolic natural variation, plant-to-plant variability seems to be larger than between-accession variance. If a broad range of metabolites are of interest, it is important to know the biological variability that is exhibited by most metabolites. If only a small subset of the non-targeted analysis is in research focus, it will be sufficient to deal with the biological variability of a certain substance class.

### 2.2. Plant-to-Plant Variability in Secondary Metabolism Is Substance-Class-Dependent, but Not Accession-Specific

**Figure 3:**
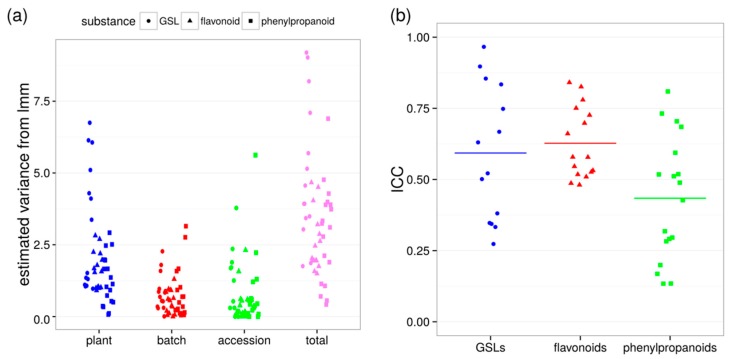
Biological variability of annotated secondary metabolites. (**a**) Variances for plant, batch and accession were estimated with a linear mixed model (lmm), dot—variance of one metabolite; (**b**) ICCs for glucosinolates (GSLs), flavonoids, and phenylpropanoids, dot—ICC of one metabolite, bar—mean ICC for substance class.

A difficulty in non-targeted metabolomics is the assignment of the measured features to metabolites and their potential role in pathways in a living system. To facilitate the interpretation of plant-to-plant variability, three sets of annotatable compounds were quantified by integrating peak areas of the extracted ion chromatograms and analysed for their variances at each level (Table S1). In Figure 3, GSLs, flavonoids, and phenylpropanoids are indicated by circles, triangles, and squares, respectively. GSLs were the substance class with the highest plant-to-plant variability (σ^2^_plant_ = 3.16, Figure 3a left, circles) compared to flavonoids and phenylpropanoids. They also showed a large deviation of the single metabolite plant variance from the mean of the substance class. Similarly, σ^2^_total_ = 5.03 was highest for GSLs in the comparison to flavonoids (σ^2^_plant_ = 1.63, σ^2^_total_ = 2.60) and phenylpropanoids (σ^2^_plant_ = 1.24, σ^2^_total_ = 2.88).

With the current experimental setup of four plants in three batches for a total of 12 plants per accession, the minimal detectable log fold-change to distinguish between two accessions is 3.94, 2.97 and 3.24 for glucosinolates, flavonoids, and phenylpropanoids, respectively, with a power of 0.8 and a significance level of 0.05. However, plant-to-plant variability needs to be interpreted in the context of total variance to find out at which experimental level the main observation is made. If σ^2^_plant_ ≈ σ^2^_total_, nearly all of the total variance would be caused by plant-to-plant variability and a large number of plants would be required to analyse effects beyond this experimental level, i.e., between accessions. If σ^2^_plant_/σ^2^_total_ ≈ 0, it would be sufficient to use one plant per accession. Glucosinolates and phenylpropanoids show a large range of ICCs. For flavonoid metabolites, the ICCs are rather high but similar for all analysed members of the substance class (Figure 3b). Hence, calculations with the mean ICCs like above will provide sufficient power for analyses of flavonoids, but not for all metabolites of the classes glucosinolates and phenylpropanoids.

A set of primary metabolites was also analysed for their plant-to-plant variability (Table S2) but, in comparison to secondary metabolism, the ICC distributions of carbohydrates, organic acids, amino acids, and phosphates covered a large range (Figure S3). As expected, the primary metabolism is more stable than secondary metabolism, the latter showing substance-class specific ICC distributions.

Until here, we assumed all accessions to have equal variances at the plant and batch level. In addition, we analysed if the accessions differ with regard to their plant-to-plant variability. For this purpose, linear mixed models were applied to estimate the variances of secondary metabolites for each accession separately. As shown in Figure S4, there are no clear highly and lowly variable accessions across the measured substance classes. However, Edi-0 showed relatively low ICCs for GSLs and flavonoids. Hi-0 and Sf-2 showed higher ICCs for all three compound classes.

In our analysis, taking the ICCs of secondary metabolite classes into consideration seems to be more important than the selection of accessions.

## 3. Discussion

Our study investigated natural variation and plant-to-plant variability of 19 key accessions in a comprehensive metabolite profiling approach. Measuring single plant extracts prevented the irreversible information loss resulting from pooling plant material and allows to distinguish between accessions and still analyse plant-to-plant variability. Environmental variation was kept to a minimum by a randomized growth regimen and selecting plants with approximately the same vigor for analyses. Both non-targeted LC/MS ionization modes indicated a higher plant-to-plant variability than natural variation between accessions and variance due to experimental batches. Plant-to-plant variability contributed to 47%–50% of the total variance, which is higher than previously reported for one particular compound class in seeds of one accession [5]. As our total variance was the sum of plant, batch and accession variance, the ICCs referring to the sum of plant and batch variance, like in the oil seed study [5], would have been larger.

Furthermore, we chose a range of secondary and primary metabolite classes for more specific analyses. Both data sets indicated that the plant-to-plant variability had the greatest contribution to the total variance of these metabolite classes. For GSLs, flavonoids and phenylpropanoids, the means of σ^2^_batch_ and σ^2^_accession_ were in the same order of magnitude, whereas for primary metabolite sets σ^2^_accession_ was less pronounced with values one order of magnitude below σ^2^_batch_. The minimal detectable effects were quite large and impractical with the given experimental setup of three experiments with four plants each. Possible combinations of biological and technical replicates to reliably detect a smaller effect can be calculated with the implementation provided by Trutschel et al. [3]. All annotated substance classes displayed higher mean ICCs than the non-targeted data sets they were derived from. The higher the fraction of features with high ICCs, the higher the number of plants that is required to maintain the power in a statistical analysis. This should be taken into consideration for future experimental designs. Flavonoid metabolites have similar ICCs within their substance class and therefore, calculation with mean ICC of the substance class will be sufficient to obtain reliable results for most metabolites in this class. Contrarily, GSLs and phenylpropanoids displayed a large ICC spread and require a substance-specific estimation of variance prior to future analyses. A previous study of root exudates has demonstrated that there are substance-specific differences in some metabolite classes due to alterations in the biosynthetic pathways [8]. Since some metabolites are specifically induced during stress response, they might not have been expressed in the unperturbed physiological state that was the focus of this study. The analysis of plant-to-plant variability in each accession revealed that ICC distributions are not distinct for any of the 19 accessions with the few exceptions of Edi-0, Hi-0, and Sf-2. However, our set of 19 accessions is too small to draw a general conclusion about accession-specific plant-to-plant variability and more accessions have to be analysed in future.

There are hints that biological variability converges after development [4] and upon exposure to stress factors [9,10]. A study of *Arabidopsis* plants exposed to a biotic stress factor, namely the endophytic fungus *Piriformospora indica*, showed substantial metabolic variability in untreated control samples and only a small spread of co-cultivated samples in principal component analyses. These samples were no single plant measurements but the batch variances in both sample classes were identical and thus, the observed deviation is expected to result from plant-to-plant variability [9]. Töpfer et al. [10] found that upon abiotic stress treatment, certain metabolites were robust in their abundance from plant to plant and displayed low coefficients of variation, whereas other metabolites showed larger plant-to-plant variability.

For future natural variation studies, it might be worth considering measuring single plants and make the data available for further analyses answering research questions on a different experimental level. We have provided estimated variances for selected substances in Supplementary Tables S1 and S2. Furthermore, we provide exemplary data and the functions in an R script for variance estimation in the Supplementary Folder S1 as well as data for additional substance classes in the targeted analysis in MTBLS338 in the MetaboLights repository. This knowledge can be exploited to appropriately design an experiment prior to its conduction because it may differ between a non-targeted screen and the analysis of specific substance classes.

## 4. Materials and Methods

### 4.1. Plant Cultivation

The *A. thaliana* accessions Bur-0, Can-0, Col-0, Ct-1, Edi-0, Hi-0, Kn-0, L*er*-0, Mt-0, No-0, Oy-0, Po-0, Rsch-4, Sf-2, Tsu-0, Wil-2, Ws-0, Wu-0, and Zu-0 were obtained as seeds from the European Arabidopsis Stock Centre (Nottingham, UK) and surface sterilized prior to plant cultivation. All accessions were cultivated in a hydroponic system under 8 h light and 22 °C as described previously [11] and in the protocol section of MTBLS338 with four plants in each of the three independent biological experiments. All samples were rotated in the growth chamber to minimize position effects. Primary root length and root fresh weight are given in MTBLS338. Out of 228 root samples, 210 and 222 from individual plants could be used for the GC/MS and LC/MS analysis, respectively.

### 4.2. Liquid Chromatography/Mass Spectrometry (LC/MS)

For LC/MS analysis, 40 mg root material were extracted in 200 µL 80% methanol/water (*v*/*v*) twice according to Böttcher et al. [12] and reconstituted in 30% methanol (*v*/*v*) containing 5 µM 2,4-dichlorophenoxyacetic acid as an internal standard. Upon full loop injection into an Acquitiy UPLC system (Waters, Eschborn/Germany) mounted with a HSS T3 column (100 × 1.0 mm, 1.8 µM particle size), samples were separated at a flow rate of 150 µL/min with mixtures of A (water/0.1% formic acid) and B (acetonitrile/0.1% formic acid) with a 20 min gradient: 0–1 min isocratic 95% A, 5% B; 1–16 min linear 5%–95% B; 16–18 min isocratic 95% B; 18–18.01 min linear 95%–5% B; 18.01–20 min isocratic 5% B. Eluates were ionized using an Apollo II source (Bruker Daltonics, Billerica, MA, USA) into a MicroTOF-Q I hybrid quadrupole time-of-flight mass analyzer (Bruker Daltonics) in both ionization modes with a mass range *m*/*z* 80–1000. Mass spectrometry settings were applied as previously described [11] and in the protocol section of MTBLS338.

All LC/MS runs were acquired as centroid spectra and recalibrated with lithium formate cluster ions for each measurement. Vendor .d file formats were converted into the open standard mzData with CompassXPort (Bruker Daltonics, Billerica, MA, USA).

### 4.3. Gas Chromatography/Mass Spectrometry (GC/MS)

For GC/MS analysis, 40 µL of the root extract were vacuum-evaporated and subjected to a derivatization with (1) methoxyamine hydrochloride and (2) *N*,*O*-bis(trimethylsilyl)-trifluoroacetamide as previously described [13]. Derivatized samples were injected in a splitless manner into a split/splitless inlet of an Agilent 6890N GC and a ZB-5 column (30 m × 0.25 mm, 0.25 m 95% dimethyl/5% diphenyl polysiloxane film, 10 m integrated guard column, Phenomenex, Aschaffenburg, Germany) at 230 °C. An Agilent 5975 Series Mass Selective Detector (Agilent Technologies, Waldbronn, Germany) was used to detect eluting compounds from *m*/*z* 70 to 600. Vendor file format conversion and baseline correction was performed by MetAlign [14].

### 4.4. Data Analysis

Statistical analysis was performed using R version 3.2.0 and the Bioconductor environment [15,16]. Functions are available as an R script in the Supplementary Folder S1.

#### 4.4.1. Raw Data Processing

All LC/MS data analysis was performed with the R packages XCMS and CAMERA [17,18,19]. Features were extracted with centWave (snthr = 10, ppm = 20, peakwidth = c(5,12), scanrange = c(1,3600)) and grouped (minfrac = 0.75, bw = 5, mzwid = 0.05), corrected for retention shifts and re-grouped with smaller bandwidth (bw = 2). Missing values were imputed by integration of raw data (fillPeaks) and with random numbers around the minimal intensity value across the samples.

Baseline-corrected GC/MS tags with intensities above 500 peak height were subsequently processed with TagFinder [20] and mass spectral features were grouped according to their common retention time. Clusters with at least 3 correlating tags were extracted and identified according to matching the Golm Metabolome Database [21]. In GC/MS, 15,539 tags were detected and 98 metabolites were annotated (Table S3).

All data were log-transformed to approximate a normal distribution for further statistics.

#### 4.4.2. Targeted LC/MS Analysis

For the targeted analysis, DataAnalysis 4.2 (Bruker Daltonics, Billerica, MA, USA) was used to extract ion chromatograms, deconvolute mass spectra and determine the elemental composition. Peak areas (minimum peak area = 500) of extracted ion chromatograms were integrated with QuantAnalysis 2.0 (Bruker Daltonics, Billerica, MA, USA) to quantify compound abundances with quasi-molecular ions as listed in Table S4 [11,22]. In the LC/MS measurements, 3305 peaks ESI(+) and 2730 peaks ESI(−) were detected and all together 139 compounds could be annotated.

#### 4.4.3. Variance Estimation with Linear Mixed Models

A linear mixed model (R package lme4, version 1.1-11, [23]) with accession, batch and plant as random effects was applied to log-transformed metabolite abundances to estimate variance contribution of each experimental level assuming equal variances for each accession. Linear mixed models with batch and plant as random effects were applied separately to each accession to examine accession-specific variances. Intraclass correlations (ICCs) were calculated as the ratio of σ^2^_plant_ and σ^2^_total_ according to Sampson et al. [7] and plotted as a cumulative distribution. Further analysis was constrained to known metabolites to allow for a better interpretation. The minimal detectable effect sizes were estimated with the power calculations for multilevel experiments [3].

### 4.5. Data Availability

All data sets including the targeted analyses are available from the MetaboLights repository under the accession number MTBLS338 [24].

## 5. Conclusions

This study investigated the variability in root metabolite profiles of 19 *A. thaliana* accessions. It revealed that plant-to-plant variability can be a substantial component of the overall variability in a natural variation analysis. Additionally, several selected substance classes were characterized by differing intraclass correlations. To exploit the full potential of a non-targeted metabolite profiling, single-plant measurements should be acquired and correctly integrated into the analysis. Hence, different substance classes of interest might require a customised experimental set-up.

## Supplementary Materials

Supplementary materials can be found at www.mdpi.com/1422-0067/17/9/1565/s1.

## Abbreviations

EI	electron ionization
ESI	electrospray ionization
GC/MS	gas chromatography/mass spectrometry
GSL	glucosinolate
ICC	intraclass correlation
LC/MS	liquid chromatography/mass spectrometry
