# Supplementary material for: Plant-to-Plant Variability in Root Metabolite Profiles of 19 Arabidopsis thaliana Accessions Is Substance-Class-Dependent
Source: Int J Mol Sci. 2016 Sep 16;17(9):1565. doi: 10.3390/ijms17091565 (PMC5037833; doi:10.3390/ijms17091565)
Supplement: Supplementary file 1 [file ijms-17-01565-s001.zip › ijms-141026-Supplementary Materials/ijms-141026-supplymentary.pdf]

Supplementary Materials: Plant-to-Plant Variability in Root Metabolite Profiles of 19 *Arabidopsis thaliana* Accessions Is Substance-Class-Dependent

Susann Mönchgesang, Nadine Strehmel, Diana Trutschel, Lore Westphal, Steffen Neumann and Dierk Scheel

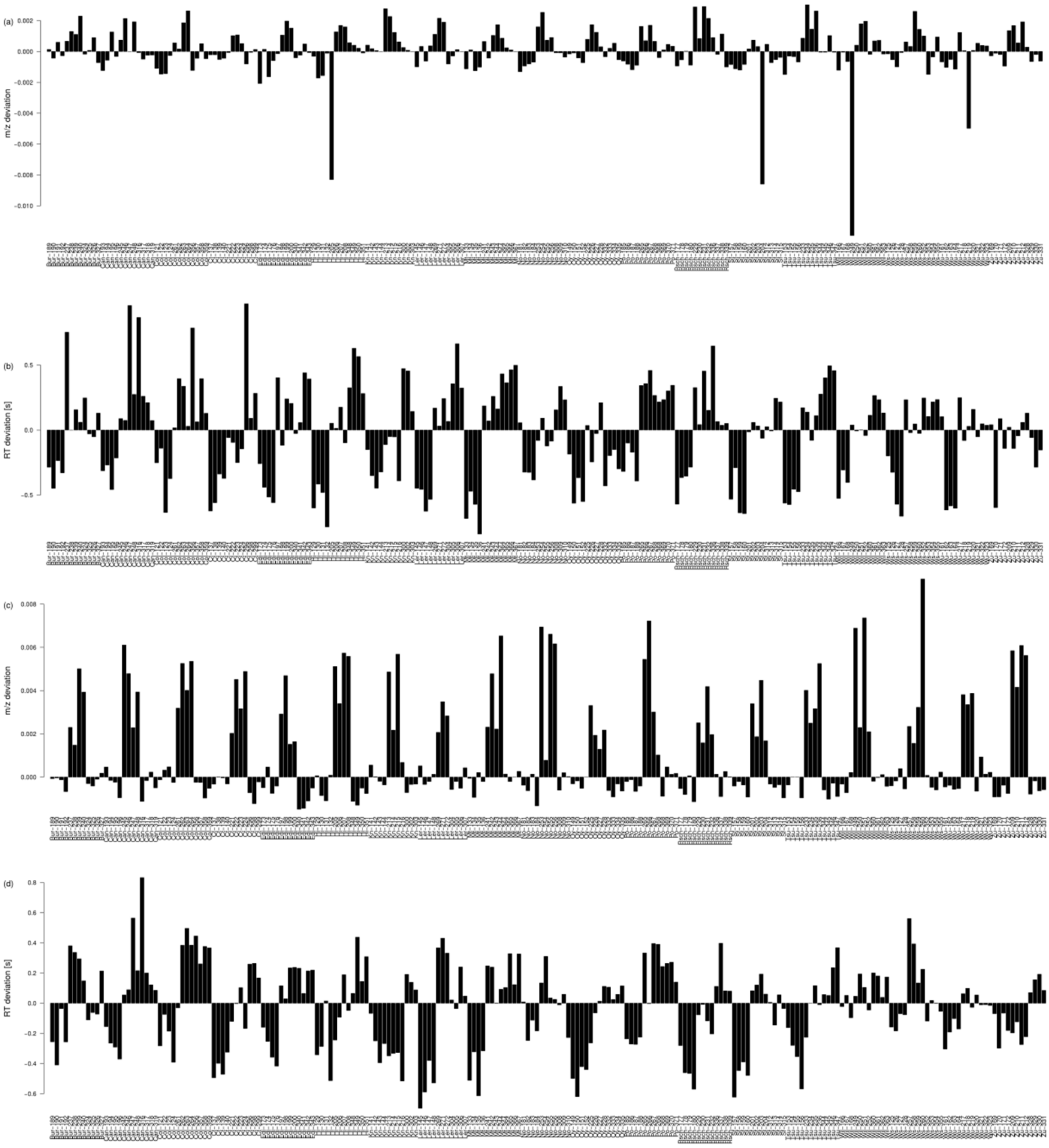

Figure S1. Quality control of liquid chromatography (LC)/electrospray ionization (ESI)(−) and ESI(+) mass spectrometry (MS) data sets. Deviation of each single plant sample in chromatography and mass analyzer, (**a**) mass-to-charge (*m*/*z*) deviation ESI(−); (**b**) retention time (RT) deviation ESI(−); (**c**) *m*/*z* deviation ESI(+); (**d**) RT deviation ESI(+).

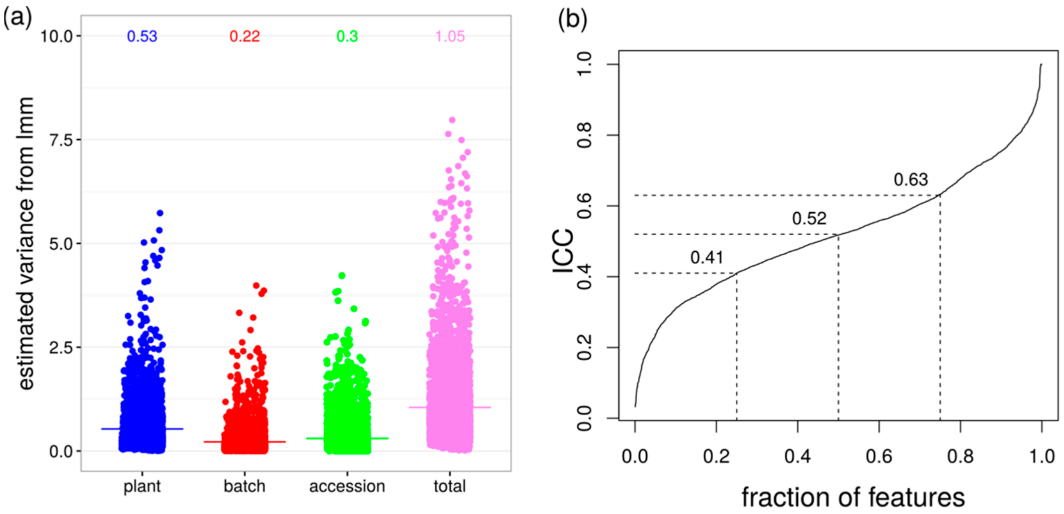

Figure S2. Variance decomposition of LC/ESI(+) MS data set. (**a**) Variances for plant, batch and accession were estimated with a linear mixed model (lmm), dot—variance of one feature, bar and number—mean variance over 3305 features; (**b**) Cumulative intraclass correlation (ICC) distribution for all features ($\sigma^2_{plant}/\sigma^2_{total}$), dotted lines indicate 25%, 50% and 75% quantiles.

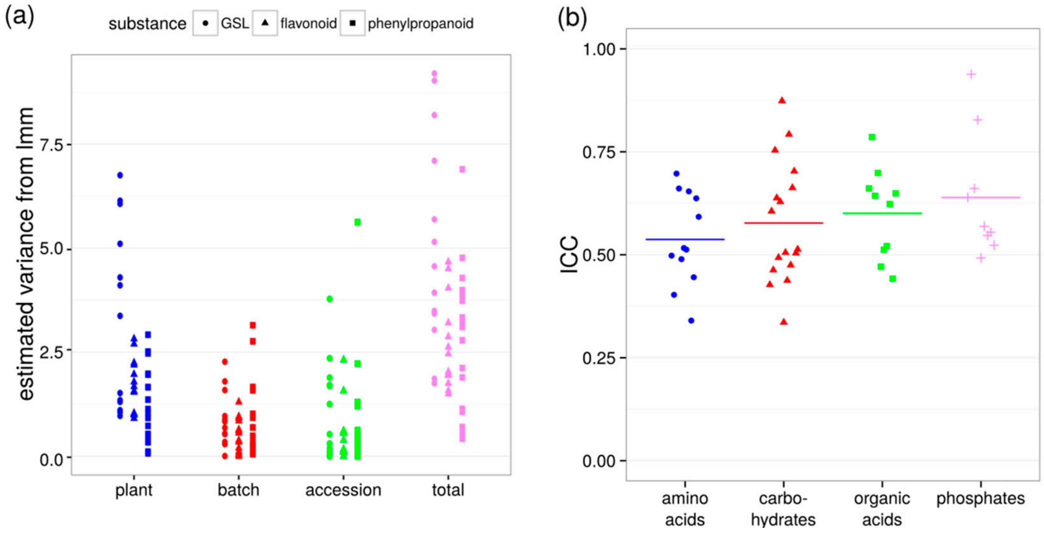

Figure S3. Biological variability of annotated primary metabolites. (**a**) Variances for plant, batch and accession were estimated with a linear mixed model (lmm), dot—variance of one metabolite; (**b**) ICCs for carbohydrates, organic acids, amino acids and phosphates, dot—ICC of one metabolite, bar—mean ICC for substance class. GSL = glucosinolate.

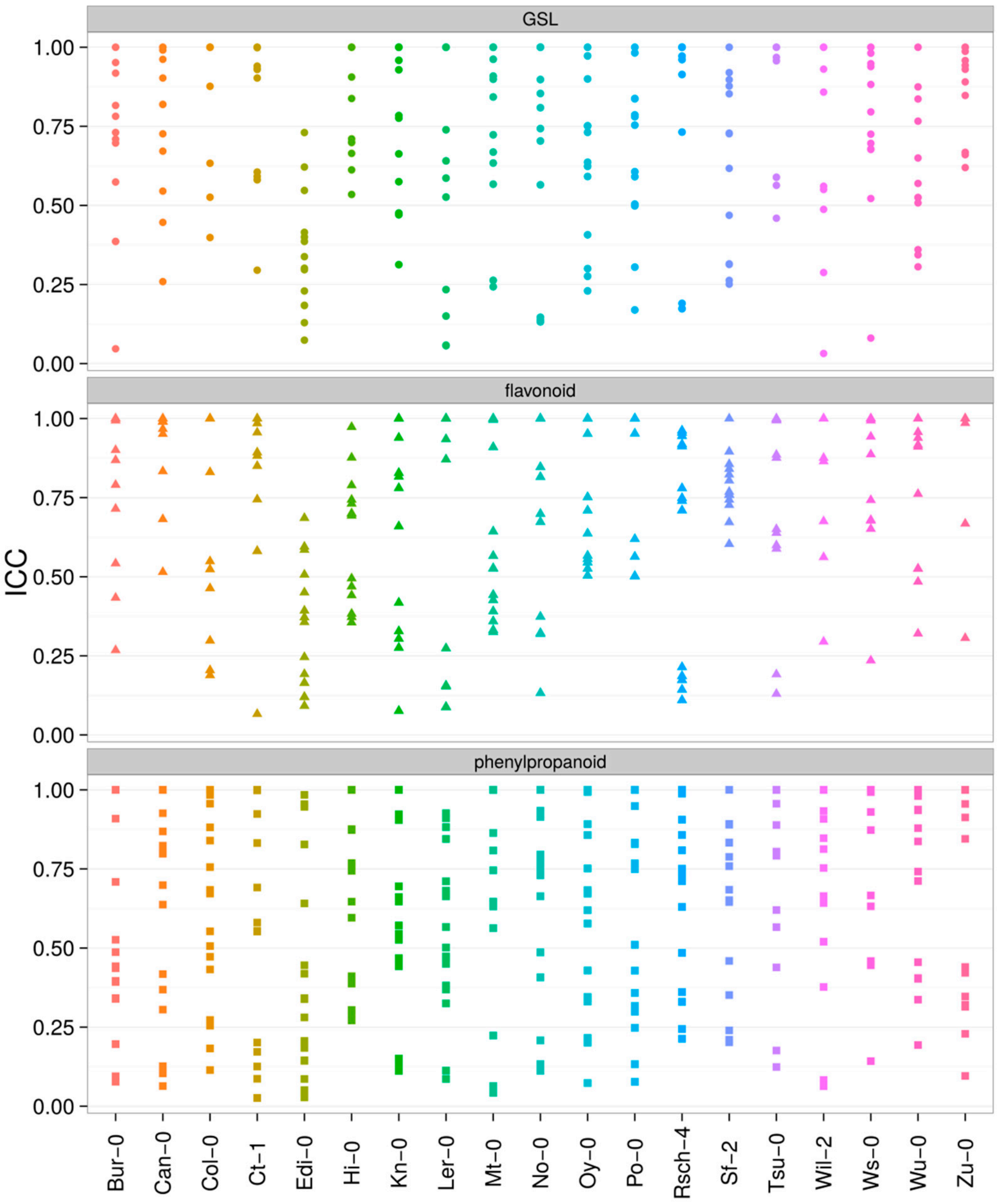

Figure S4. Accession-specific ICCs of secondary metabolites. ICCs were determined as $\sigma^2_{plant}/\sigma^2_{total}$ from 19 linear mixed models. GSL = glucosinolate.

Table S1. Estimated variances for accession, experiment and plant from a linear mixed model and intraclass correlation (ICC) for each secondary metabolite and the mean for each substance class (bold). GSL = glucosinolate, MeS = methylthio, MeSO = methylsulfinyl.

Metabolite	σ^2_{plant}	σ^2_{batch}	$\sigma^2_{accession}$	σ^2_{total}	ICC
3-MeS-Propyl-GSL	1.108	0.348	0.301	1.757	0.631
4-MeS-Butyl-GSL	1.054	0.293	1.688	3.035	0.347
5-MeS-Pentyl-GSL	1.351	0.866	1.712	3.929	0.344
4-MeSO-Butyl-GSL	1.075	0.967	1.890	3.932	0.273
5-MeSO-Heptyl-GSL	1.519	0.687	2.356	4.563	0.333
6-MeSO-Hexyl-GSL	1.307	1.592	0.532	3.431	0.381
7-MeS-Heptyl.GSL	6.139	1.799	1.256	9.194	0.668
7-MeSO-Heptyl-GSL	4.296	0.549	0.305	5.150	0.834
8-MeS-Octyl-GSL	6.751	2.273	0.000	9.024	0.748
8-MeSO-Octyl-GSL	5.106	0.533	0.052	5.691	0.897
1-MeO-I3M-GSL	4.109	0.305	3.781	8.196	0.501
4-MeO-I3M-GSL	3.374	0.009	0.110	3.492	0.966
I3M-GSL	6.065	0.836	0.194	7.096	0.855
6-MeS-Hexyl-GSL	0.972	0.892	0.000	1.864	0.522
Quercetin-3-*O*-Deoxyhex-Hex-7-*O*-Deoxyhex	2.245	0.852	1.572	4.669	0.481
Kaempferol-(Deoxyhex)$_2$	1.677	0.197	0.157	2.031	0.826
Kaempferol-(Deoxyhex)$_2$ Hex a	1.540	0.047	0.388	1.975	0.780
Kaempferol-(Deoxyhex)$_2$ Hex b	2.821	0.611	0.610	4.042	0.698
Kaempferol-(Hex)$_2$	0.992	0.956	0.000	1.948	0.509
Kaempferol-DeoxyhexHex a	0.911	0.110	0.554	1.575	0.578
Kaempferol-DeoxyhexHex b	1.788	0.560	0.115	2.463	0.726
Kaempferol-Deoxyhex a	1.024	0.921	0.002	1.946	0.526
Kaempferol-Hex	1.038	0.915	0.000	1.953	0.531
Naringenin-Hex	0.990	0.384	0.124	1.497	0.661
Quercetin-(Deoxyhex)$_2$ Hex a	2.697	0.343	0.167	3.208	0.841
Quercetin-(Deoxyhex)$_2$ Hex b	2.194	0.000	2.314	4.508	0.487
Quercetin-(Hex)$_2$	1.014	0.642	0.096	1.752	0.579
Quercetin-DeoxyhexHex a	1.568	1.305	0.000	2.872	0.546
Quercetin-DeoxyhexHex b	1.970	0.061	0.593	2.624	0.751
Coniferin	0.364	0.228	0.111	0.703	0.518
Syringin	0.337	0.226	0.576	1.139	0.296
S(8-8)S	1.658	1.584	0.000	3.242	0.511
Lariciresinol	1.981	0.926	0.428	3.335	0.594
Lariciresinol-Hex	1.961	0.267	0.556	2.785	0.704
Esculetin	2.472	1.666	0.628	4.766	0.519
Scopoletin	1.036	0.493	0.591	2.119	0.489
Esculin	0.732	0.114	0.223	1.068	0.685
Scopolin	1.663	1.020	1.210	3.893	0.427
Scopoletin Hex-Pent	0.071	0.051	0.300	0.423	0.168
Scopoletin Benzoyl-Hex-Pent	0.111	0.113	0.334	0.558	0.199
Unknown oligolignol	2.920	0.691	0.380	3.991	0.732
G(8-*O*-4)FA-Sulfate	0.922	0.347	5.623	6.892	0.134
G(8-5)FA	1.362	0.696	2.225	4.283	0.318
G(8-5)FA-Sulfate	0.536	0.057	1.302	1.895	0.283
G-Hex	2.514	0.144	0.449	3.106	0.809
Coniferyl alcohol(8-*O*-4)sinapoylmalate	1.134	2.762	0.000	3.896	0.291
1-*O*-Sinapoyl-beta-glucose	0.502	3.148	0.085	3.735	0.134

Table S1. *Cont.*

Metabolite	σ^2_{plant}	σ^2_{batch}	$\sigma^2_{accession}$	σ^2_{total}	ICC
**GSLs**	3.159	0.853	1.013	5.025	0.593
**Flavonoids**	1.631	0.527	0.446	2.604	0.635
**Phenylpropanoids**	1.238	0.807	0.834	2.879	0.434

Table S2. Estimated variances for accession, experiment and plant from a linear mixed model and ICC for each primary metabolite and the mean for each substance class (bold). TMS = trimethylsyliated, MEOX = methoxymated, MP = main product, BP = by-product, GABA = γ-aminobutyric acid.

Metabolite	σ^2_{plant}	σ^2_{batch}	$\sigma^2_{accession}$	σ^2_{total}	ICC
A122001_Valine-2TMS	0.063	0.043	0.020	0.127	0.498
A129002_Leucine-2TMS	0.086	0.084	0.006	0.176	0.489
A133001_Glycine-3TMS	0.151	0.108	0.033	0.293	0.516
A138001_Serine-3TMS	0.140	0.094	0.039	0.273	0.513
A138005_Alanine-3-cyano-2TMS	0.263	0.079	0.060	0.403	0.654
GABA-2TMS	0.033	0.017	0.046	0.096	0.340
A140001_Threonine-3TMS	0.158	0.140	0.056	0.354	0.445
A144003_Aspartic acid-2TMS	0.132	0.075	0.000	0.208	0.637
A144001_beta-Alanine-3TMS	0.162	0.096	0.016	0.273	0.592
A153002_Pyroglutamic acid-2TMS	0.228	0.243	0.095	0.567	0.403
A163001_Glutamic acid-3TMS	0.515	0.152	0.071	0.739	0.697
A164001_Phenylalanine-2TMS	0.677	0.347	0.000	1.024	0.661
D(-)-Erythrose-tris(trimethylsilyl)ether,methyloxime (syn)	0.157	0.048	0.263	0.467	0.336
A154001_Erythronic acid-4TMS	0.047	0.030	0.017	0.093	0.505
Arabinonic acid lactone-3TMS	0.033	0.033	0.010	0.076	0.438
A165001_Xylose-1MEOX-4TMS-MP	0.136	0.036	0.000	0.171	0.792
A177001_Ribonic acid-5TMS	0.080	0.057	0.031	0.168	0.475
A179001_Arabinonic acid-5TMS	0.259	0.097	0.034	0.391	0.663
Talofuranose-5TMS	0.054	0.023	0.000	0.077	0.703
A187002_Fructose-1MEOX-5TMS-MP	0.082	0.080	0.000	0.162	0.504
A188004_Fructose-1MEOX-5TMS-BP	0.113	0.107	0.000	0.219	0.513
A188006_Glucopyranoside-1-O-methyl-alpha-4TMS	0.072	0.093	0.003	0.168	0.427
A189002_Glucose-1MEOX-5TMS-MP	0.009	0.005	0.000	0.015	0.606
A191001_Glucose-1MEOX-5TMS-BP	0.107	0.115	0.009	0.230	0.463
A19900_Galactonic acid-6TMS	0.067	0.020	0.002	0.089	0.754
A200001_Gluconic acid-6TMS	0.242	0.137	0.000	0.379	0.638
A207012_Glucosamine-N-acetyl-1MEOX-4TMS	0.117	0.067	0.054	0.237	0.493
A264001_Sucrose-8TMS	0.027	0.016	0.000	0.043	0.629
A274001_Maltose-1MEOX-8TMS-MP	0.359	0.052	0.000	0.410	0.874
A105001_Lactic acid-2TMS	0.109	0.030	0.000	0.139	0.786
A135003_Glyceric acid-3TMS	0.907	0.278	0.225	1.409	0.643
Methylsuccinic acid-2TMS	0.069	0.012	0.018	0.099	0.699
A149001_Malic acid-3TMS	0.138	0.125	0.029	0.292	0.471
A156001_Threonic acid-4TMS	0.117	0.081	0.031	0.228	0.512
A158004_Glutaric acid-2-oxo-1MEOX-2TMS-MP	0.187	0.143	0.029	0.359	0.521
A161003_Glutaric acid-3-hydroxy-3-methyl-3TMS	0.459	0.271	0.006	0.736	0.623
A182004_Citric acid-4TMS	0.185	0.152	0.081	0.419	0.442
A205001_Hexadecanoic acid-1TMS	0.016	0.009	0.000	0.025	0.649
A225002_Octadecanoic acid-1TMS	0.019	0.010	0.000	0.028	0.661
A119001_Phosphoric acid monomethyl ester-2TMS	0.049	0.028	0.000	0.077	0.639
A129001_Phosphoric acid-3TMS	0.001	0.000	0.000	0.001	0.938
A177002_Glycerol-3-phosphate-4TMS	0.129	0.066	0.000	0.195	0.661
A177014_Ethanolaminephosphate-4TMS	0.425	0.026	0.063	0.513	0.827
A218002_Glycerophosphoglycerol-5TMS	0.135	0.068	0.072	0.275	0.492
A233002_Glucose-6-phosphate-1MEOX-6TMS-MP	0.120	0.090	0.001	0.210	0.569
A235002_Glucose-6-phosphate-1MEOX-6TMS-BP	0.115	0.088	0.007	0.211	0.547
A243001_myo-Inositol-1-phosphate-7TMS	0.051	0.038	0.003	0.092	0.555
A249002_Inositol-1-phosphate-myo-7TMS	0.043	0.014	0.025	0.081	0.523
A177002_Glycerol-3-phosphate-4TMS	0.129	0.066	0.000	0.195	0.661
A177014_Ethanolaminephosphate-4TMS	0.425	0.026	0.063	0.513	0.827
A218002_Glycerophosphoglycerol-5TMS	0.135	0.068	0.072	0.275	0.492
A233002_Glucose-6-phosphate-1MEOX-6TMS-MP	0.120	0.090	0.001	0.210	0.569
A235002_Glucose-6-phosphate-1MEOX-6TMS-BP	0.115	0.088	0.007	0.211	0.547
**Amino acids**	0.217	0.123	0.037	0.378	0.537
**Carbohydrates**	0.115	0.060	0.025	0.200	0.577
**Organic acid**	0.221	0.111	0.042	0.374	0.601
**Phosphates**	0.119	0.046	0.019	0.184	0.639
